# A prospective longitudinal study shows putamen volume is associated with moderate amphetamine use and resultant cognitive impairments

**DOI:** 10.1093/psyrad/kkab001

**Published:** 2021-03-18

**Authors:** Keith M Kendrick, Joerg Daumann, Daniel Wagner, Philip Koester, Marc Tittgemeyer, Qiang Luo, Euphrosyne Gouzoulis-Mayfrank, Benjamin Becker

**Affiliations:** The Clinical Hospital of Chengdu Brain Science Institute, Key Laboratory for Neuroinformation, Center for Information in Medicine, School of Life Science and Technology, University of Electronic Science and Technology of China, Chengdu, China; Department of Psychiatry and Psychotherapy, University of Cologne, Germany; Department of Psychiatry and Psychotherapy, University of Cologne, Germany; Department of Psychiatry and Psychotherapy, University of Cologne, Germany; Max-Planck Institute for Neurological Research, Cologne, Germany; Institute of Science and Technology for Brain-Inspired Intelligence, Ministry of Education Key Laboratory of Computational Neuroscience and Brain-Inspired Intelligence, Fudan University, Shanghai, PR China; LVR Clinics of Cologne, Cologne, Germany; The Clinical Hospital of Chengdu Brain Science Institute, Key Laboratory for Neuroinformation, Center for Information in Medicine, School of Life Science and Technology, University of Electronic Science and Technology of China, Chengdu, China

**Keywords:** amphetamines, brain volume, dorsal striatum, MDMA, prospective, stimulants

## Abstract

**Background:**

Amphetamine-type stimulants (ATS) have become a critical public health issue. Animal models have indicated a clear neurotoxic potential of ATSs. In humans, chronic use has been associated with cognitive deficits and structural brain abnormalities. However, cross-sectional retrospective designs in chronic users cannot truly determine the causal direction of the effects.

**Objective:**

To prospectively determine effects of occasional ATS use on cognitive functioning and brain structure.

**Methods:**

In a prospective longitudinal study design, cognitive functioning and brain structure were assessed at baseline and at 12-month follow-up in occasional ATS users (cumulative lifetime use <10 units at baseline).

**Results:**

Examination of change scores between the initial examination and follow-up revealed declined verbal memory performance and putamen volume in users with high relative to low interim ATS exposure. In the entire sample, interim ATS use, memory decline, and putamen volume reductions were strongly associated.

**Conclusions:**

The present findings support the hypothesis that ATS use is associated with deficient dorsal striatal morphology that might reflect alterations in dopaminergic pathways. More importantly, these findings strongly suggest that even occasional, low-dose ATS use disrupts striatal integrity and cognitive functioning.

## Introduction

Increasing rates of recreational amphetamine-type stimulant (ATS) use, predominately illicitly produced amphetamine (AMPH) and 3,4-methylenedioxymethamphetamine (MDMA, “Ecstasy”) and of ATS users seeking treatment indicate that ATSs have become a major health problem (UNODC, 2011; 2014). In terms of prevalence rates, ATS is second only to cannabis (UNODC, 2011), with recreational use among, often socially well-integrated, young adults being the most typical pattern (Gouzoulis-Mayfrank and Daumann, 2009) . During the last decades converging evidence from different animal models indicates a neurotoxic potential of ATSs (Aguilar *et al*., 2020; Parrott, 2013; Moratalla *et al*., 2017). These animal studies have shown that the experimental application of varying dosage regimens of MDMA and amphetamines lead to long-term neurotoxic effects in rodent and nonhuman primate models, as indicated by a range of brain morphological and neurochemical indices (for an overview, see e.g. Moratalla *et al*., 2017). However, the key question as to whether human ATS users may suffer from similar neurotoxic brain lesions remains unanswered.

Convergent evidence from animal models and meta-analyses covering neuroimaging studies in human drug users suggest that prolonged drug use is associated with structural and functional adaptations in limbic-striato-prefrontal circuits of the brain (Everitt and Robbins, 2016; Klugah-Brown *et al*., 2020; Ersche *et al*., 2013). Accumulating evidence from human studies suggests that the chronic use of ATS is associated with altered brain morphology, particularly deficient gray matter (GM) integrity in limbic-striato-prefrontal brain networks, as well as subtle yet consistently observed, deficits in cognitive and emotional functions that have been associated with this circuitry (Gouzoulis-Mayfrank and Daumann, 2009; Wagner *et al*., 2013; Ersche *et al*., 2013; Mackey and Paulus, 2013; Parrott, 2015). However, most human findings are based on cross-sectional studies in the sub-group of chronic, often dependent, users of the more-addictive amphetamine compound methamphetamine (MA), also known as “crystal-meth”. Due the retrospective design and the lack of baseline data, these studies do not allow a separation of specific effects of ATS use, such as potential neurotoxic effects or addiction-related brain-plastic adaptations, from alterations that precede, or promote, the onset of use. Only longitudinal designs that control for baseline differences can truly determine whether the neuropsychological or neuroanatomical differences in ATS users are a result of drug use or a predisposing factor (Taylor *et al*., 2013).

Using sophisticated sampling strategies in cross-sectional study designs, which also include more appropriate control groups and prospective designs, we and others have begun to disentangle the contribution of predisposing and drug-associated factors in brain-structural abnormalities observed in ATS users (Daumann *et al*., 2011; Ersche *et al*., 2012; Becker *et al*., 2015). Findings from these studies suggest that GM alterations in regions associated with emotional and cognitive control, particularly the amygdala, the anterior cingulate, and adjacent medial prefrontal regions before the onset of ATS use may represent reliable brain-structural vulnerability markers for increased risk to develop escalating use and potential addiction. However, studies with longitudinal-designs specifically focusing on brain-structural effects of ATS users while controlling for baseline abnormalities is rare.

The assessment of brain-structural changes in longitudinal designs has additionally been hampered by methodological issues. Traditional longitudinal voxel-based morphometry (VBM) (Ashburner and Friston, 2000) analyses use simple intra-participant registration approaches and asymmetric processing that bias the estimation of longitudinal changes (Ashburner and Friston, 2011; Thompson and Holland, 2011). More recent developments in longitudinal VBM techniques, such as group-wise intra-participant models (symmetric approaches) that combine rigid-body and diffeomorphic (Ashburner and Friston, 2011) registration and correction for inhomogeneity artifacts (Ashburner and Ridgway, 2012) have enabled researchers to evaluate brain-structural changes with more appropriate statistical models and accordingly a higher sensitivity for longitudinal changes.

Against this background, we applied the optimized VBM machinery to a longitudinal brain-structural dataset acquired in occasional ATS users with only minimal ATS exposure at study inclusion (cumulative lifetime use <10 units of ATS) to specifically examine the long-term effects of ATS use on brain structure while controlling for baseline differences and other known confounders in this field (e.g. co-use of other drugs, particularly cannabis; Gouzoulis-Mayfrank and Daumann, 2006). To this end brain structure, cognitive functioning and interim drug use were re-assessed after a follow-up period of 12-months. Using a data-driven clustering approach, users with low (LOW) and high (HIGH) ATS use during follow-up were identified. Next, cognitive domains and brain regions with differential between-group changes during follow-up were explored using a correlational approach to take advantage of the entire sample of *n* = 17 in examining ATS use-associated functional and structural changes.

## Materials and methods

### Participants

Participants in the present study were a sub-group of a larger research project and their baseline data had been used for cross-sectional brain-structural comparisons (Daumann *et al*., 2011; Becker *et al*., 2015). The main inclusion criterion at baseline was occasional (ATS use >1 occasion), but very limited use of ATS (cumulative lifetime use of <10 units of ATS). In line with previous studies (Daumann *et al*., 2011; Becker *et al*., 2015) units were defined on the basis of typical quantities that the MDMA and amphetamine are supplied in (one unit MDMA = 1 tablet; one unit amphetamine = 1 g). In addition, the following exclusion criteria were applied: (i) lifetime use of any other illicit psychotropic substances on more than five occasions (except for cannabis, which is widely used among recreational ATS users), (ii) history of alcohol abuse or dependence according to DSM-IV criteria, (iii) regular medication (once or more a week, except for contraceptives) or current use of psychotropic substances (in the 7 days before the examination, exceptions were cannabis, tobacco), (iv) current or history of a neurological or psychiatric disorder according to Axis I or II DSM-IV criteria, (v) any other general medical condition or history of traumatic brain injury with loss of consciousness or amnesia, (vi) left-handedness, (vii) unable to give informed consent, (viii) age below 18 years, (ix) childhood diagnosis of attention-deficit hyper-activity disorder, (x) pregnancy, and (xi) MRI contraindications. Importantly, a previously published cross-sectional comparison with drug-naïve participants revealed no brain-structural alterations in the group of occasional ATS users (Daumann *et al*., 2011). In addition, cognitive functioning as well as a range of potential confounders, including use of other licit and illicit drugs, psychopathology, cognitive functioning, and urine, as well as hair samples to validate drug-use patterns were assessed (details see Wagner *et al*., 2013; Becker *et al*., 2013). Follow-up brain-structural data could be assessed in *n* = 19 from the *n* = 42 participants that were included during the baseline assessments. The data acquisition was discontinued after the initial 19 re-assessments due to a change of personnel in the study team. Cognitive performance was assessed using a battery of validated neurocognitive tests assessing speed of information processing, cognitive inference and flexibility as well as verbal and visual long- and short-term memory (Wagner *et al*., 2013). Following a written description of the experimental protocols eligible individuals provided written informed consent. The study protocols had full ethical approval by the Medical Faculty of the University of Cologne and were in accordance with the latest revision of the Declaration of Helsinki.

### Procedures

At baseline, 42 occasional ATS users were enrolled in the cross-sectional study (for details, see Daumann *et al*., 2011). After baseline assessment of brain structure, drug use, cognitive performance and potential confounders participants were followed to re-assess brain structure, cognitive functioning and interim ATS use during a 12-months follow-up interval. At follow-up brain structure could be re-assessed in a total of *n*  = 19 participants. During the initial screening Axis I and II disorders were assessed via a structured interview according to DSM-IV criteria, childhood ADHD was retrospectively assessed using the Wender Utah Rating Scale (Ward et al., 1993) and drug use for ATS and other prevalent psychotropic substances was assessed using a structured interview. Moreover, potential confounders were assessed in the domains of neuropsychological functioning, non-verbal intelligence, and overall psychological distress (Global Severity Index from the Symptom Checklist-90-R, SCL90R), as well as alcohol and tobacco use. Self-reported substance use patterns were further validated by randomly taken hair samples and drug urine screens.

### Cognitive test battery

#### Auditiv-Verbaler Lerntest AVLT

Verbal declarative memory performance was examined using the German version of the Rey Auditory Verbal Learning Test (Rey, 1964; Auditiv-Verbaler Lerntest (AVLT), Heubrock, 1992). The test assesses verbal declarative memory performance in the domains of immediate recall, total acquisition performance across five trials, recall after interference, loss after interference, and recognition after an interval of 30 minutes.

#### Lern- und Gedächtnistest LGT 3

Visual paired associates learning was assessed by means of a subtest of the Lern- und Gedächtnistest (LGT) (Baeumler, 1974). This subtest presents figures composed of a logo and surrounding frame presented for 60 seconds. Participants are required to select the correct logo-frame combination from four options, immediately after the presentation (immediate recall) and after an interval of 1 hour (delayed recall).

#### Digit-Span-Backward

The Digit-Span-Backward test from the Hamburg-Wechsler-Intelligenztest für Erwachsene (HAWIE-R) (Tewes, 1994), a German version of the Wechsler Intelligence Test (WAIS) (Wechsler, 2008) was used to assess working memory performance. Participants are presented with an auditory presented sequence of digits and are required to recall the digits immediately in reverse order.

#### Digit symbol test

This subtest from the WAIS (Wechsler, 2008) (German Version HAWIE-R) measures speed of information processing by means of the presentation of nine digit-symbol pairs (e.g. 1/-, 2/_, … 7/L, 8/X, 9/= ) followed by the presentation of a list of 93 digits. Participants have to write down the corresponding symbol for each digit as fast as possible. The total number of correct symbols written down within 90 seconds is derived as measure of speed of information processing.

#### Stroop task

An extensively validated version of the classical Stroop task (German Version, Farbe-Wort-Interferenztest (Stroop, 1935)) was used to assess performance in the domain of cognitive interference/inhibition processing. Performance is assessed in the domains of speed of performance, corrected errors, and uncorrected responses for reading color names, as well as color rectangles and color names in different colored inks (inference condition).

#### Trail-making test

Mental flexibility was assessed by the trail-making test, which requires participants to connect circles numbered from 1 to 25 (Part A) and numbers (1–13) and letters (A–L) alternatively (Part B). Response times are derived as a measure of performance.

#### Raven standard progressive matrices

General non-verbal intelligence at baseline was assessed using the Raven Standard Progressive Matrices (Raven *et al*., 1998).

### MRI data acquisition and analysis approach

High-resolution brain-structural MRI data was collected on a 3 Tesla Magnetom Tim Trio system equipped with a standard quadrature head coil (flip angle = 18^o^, repetition time = 1930 ms, echo time = 5.8 ms, slice thickness = 1.25 mm, voxel size = 1.0 × 1.0 × 1.25 mm). Analyses of the longitudinal data were carried out using optimized segmentation protocols and the new longitudinal registration module in SPM12 (Ashburner and Ridgway, 2012). The longitudinal registration approach is based on a generative modeling framework that combines rigid-body registration diffeomorphic warping with a differential intensity non-uniform correction on the level of a within-participants template (Ashburner and Ridgway, 2012). In line with previous studies (Wagner *et al*., 2013; Schilt *et al*., 2007), ATS use-associated changes in cognitive functioning were assessed by means of change scores between baseline and follow-up. In accordance with this approach, effects on GM volume were assessed using individual differential GM maps as produced by the longitudinal analysis toolbox (baseline vs. follow-up) that reflect individual GM changes between baseline and follow-up assessment. The data were subsequently smoothed with a full-width at half-maximum isotropic Gaussian kernel of 10 mm. Groups were directly compared using independent *t*-tests, comparing the differential changes between the groups. To determine the direction of the longitudinal changes, parameter estimates for the identified regions were extracted from the differential maps (baseline vs. follow-up). To increase the sensitivity to detect brain-structural changes, the analyses focused on key brain structures associated with ATS use (Ersche *et al*., 2013; Mackey and Paulus, 2013; Becker *et al*., 2015), namely basal ganglia, amygdala, medial prefrontal cortex, inferior frontal gyrus, and insula, using structural regions of interest (ROI). Structural ROI were defined using the Anatomy Toolbox v.1.8 (Eickhoff *et al*., 2005) and the WFU Pickatlas Toolbox (Maldjian *et al*., 2003). Between-group differences within the a priori ROI were computed using a threshold of *P* < 0.05 (family-wise error (FWE) corrected). Results were thresholded at a FWE corrected *P* < 0.05. For the analyses, variables that were not normally distributed, including interim ATS use, were initially log-transformed to achieve a normal distribution.

## Results

Based on automatized standard quality assessments of MRI data, one participant was excluded from all further analyses. Participants had used a mean of 7.72 (SD 8.99, range 0–27) units of ATS during the follow-up period. One user reported having used 74.2 units of ATS during follow-up and was excluded as outlier from all further analyses (*z* = 3.51). Based on the reported log-transformed ATS use during follow-up data-driven *k*-means clustering with squared Euclidean distance revealed two separate sub-groups of users with low (LOW, *n* = 11) and high ATS use (HIGH, *n* = 8). Users in the LOW (*n* = 10) group had used a mean of 1.45 (SD 1.27, range 0–3.50) units of ATS, whereas those in the HIGH (*n* = 7) group had used a mean of 16.69 (SD 7.33, range 8.80–18.20) units during the follow-up (paired *t*-test, *t* = −6.52, degrees of freedom (df) = 15, *P* < 0.001). With respect to the maximum dosage of MDMA and amphetamine, respectively, used per single occasion during the interim interval the users in the HIGH group reported maximum dosages of a mean of 2.37 (SD 1.32, range 1.25–5) ecstasy pills and a maximum dosage of a mean of 0.83 g (SD 0.51, range 0.1–1.5) amphetamines. Importantly, the LOW and HIGH groups did not show differences on a range of potential confounders at baseline, including socio-demographics and pre-baseline drug use compared to follow-up, and including days between the scanning sessions and interim cannabis use (Table 1).

**Table 1:** Demographic and drug-use characteristics of the groups.

	LOW (*n* = 10)	HIGH (*n* = 7)	*P* value
**At study inclusion**			
Age	24.50 (±5.38)	22.29 (±5.52)	0.42
Education (years)	15.45 (±2.92)	13.32 (±2.56)	0.14
General intelligence (Raven, errors)	6.40 (±4.35)	7.57 (±8.67)	0.72
No of cigarettes/day	6.30 (±6.83)	10.07 (±7.07)	0.28
Years of tobacco use	4.95 (±6.08)	5.14 (±4.22)	0.94
No alcohol drinks/week	8.00 (±1.82)	8.28 (±0.76)	0.71
Age cannabis use onset	15.30 (±2.41)	16.29 (±6.62)	0.67
Frequency of cannabis use (days/month)	16.15 (±11.31)	16.14 (±14.61)	0.99
ATS cumulative (units)	5.10 (±2.60)	6.10 (±1.88)	0.40
**During follow-up**			
Days between t1 and t2	447.50 (±109.95)	382.28 (±54.13)	0.17
ATS cumulative (units)	1.45 (±1.27)	16.68 (±7.33)	<0.001**
No of cigarettes/day	7.40 (±9.03)	7.29 (±7.95)	0.97
No alcohol drinks/week	8.60 (±1.35)	7.00 (±1.83)	0.06
Frequency of cannabis use (days/month)	15.15 (±11.41)	16.00 (±10.96)	0.88
Cannabis cumulative (gram)	84.36 (±83.51)	125.14 (±111.8)	0.40

**Figure 1:**
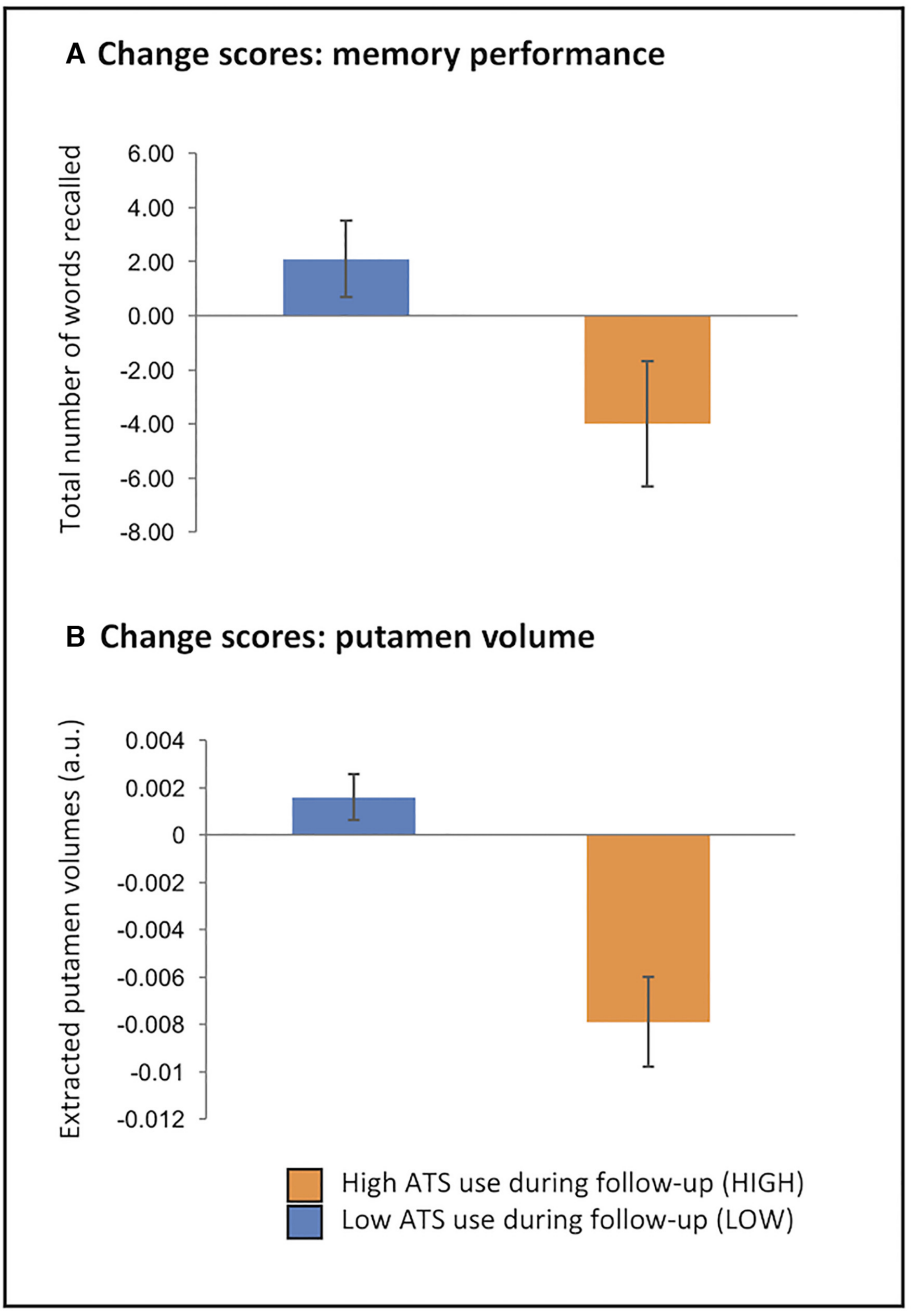
Memory performance and putamen GM change scores for the groups. Change scores (baseline vs. follow-up) from verbal memory performance (**A**) and putamen GM (**B**). Users with higher ATS use (HIGH) during follow-up demonstrated significant performance and putamen GM loss relative to users with low ATS use (LOW) during follow-up.

**Figure 2:**
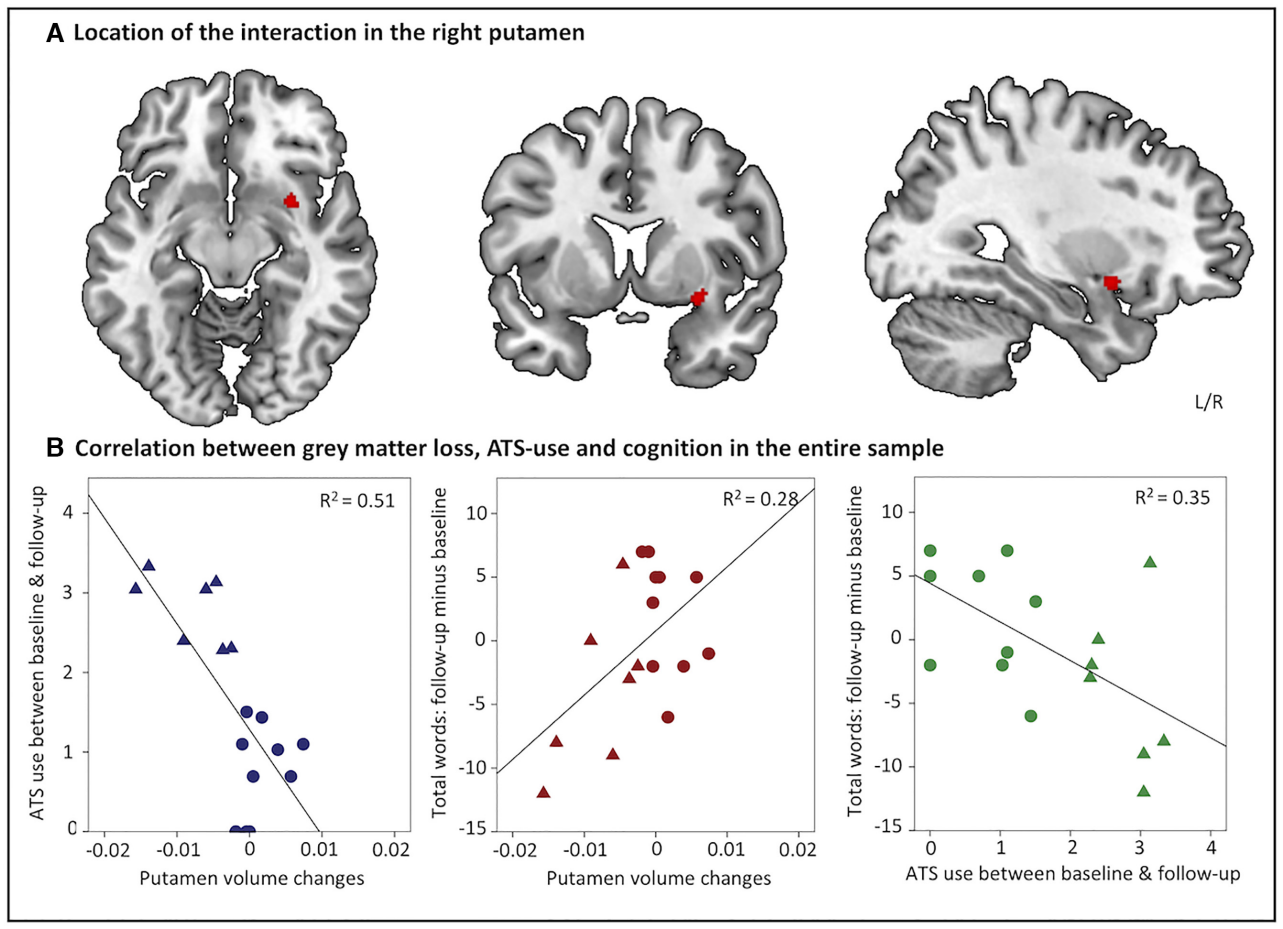
Comparison of gray matter and associations with ATS use and verbal memory performance decline, respectively. The differential changes between the HIGH and LOW group were located in the dorsal right striatum (putamen) (**A**). A higher putamen gray matter loss was associated with higher ATS use and a higher cognitive decline between baseline and follow-up (**B**). In addition, higher interim ATS use was associated with stronger cognitive decline (all *P* < 0.05).

Analyses of change scores revealed significant differences between the HIGH and LOW groups only in the domain of verbal memory (total number of words recalled across five trials of a word list; Rey Auditory Verbal Learning Test, RAVLT, Rey 1964) (*t* = 2.347, df = 15, *P* = 0.032). Compared to the baseline assessment users in the LOW group remembered on average 2.1 (SD = 4.5) words more at follow-up, whereas the HIGH group remembered on average 4.0 (SD = 6.13) fewer words at follow-up (Fig. 1a). The groups did not differ on change scores for other cognitive measures (all *P* > 0.07). Analyses of brain-structural data revealed a significant interaction effect in the basal ganglia located in the right putamen (*t* = 4.31, *P* < 0.05, maximum t-value at x = 30, y = 8, z = -9, coordinates provided in MNI space, Fig. 2a). Extraction of individual GM volumes from this region further revealed that this effect was driven by a significant reduction in the HIGH group (*t* = 4.07, df = 6, *P* = 0.007), whereas GM indices did not change significantly in the LOW group (*P* = 0.148) (Fig. 1b). A correlational analysis that took advantage of the entire sample revealed a significant negative association between interim ATS use and GM changes (*n* = 17, *r* = −0.72, *R*^2^ = 0.51, *P* = 0.001), indicating a direct association between the amount of interim ATS use and GM reductions in the right putamen. In addition, the change in the total number of words remembered in the RAVLT (follow-up minus baseline) significantly correlated with both GM changes in the right putamen (*n* = 17, *r* = 0.53, *R*^2^ = 0.28, *P* = 0.029) as well as the amount of interim ATS use (*n* = 17, *r* = −0.59, *R*^2^ = 0.35, *P* = 0.012), indicating that a higher loss of words recalled was associated with higher putamen decreases as well as higher ATS use during follow-up (correlations are shown in Fig. 2b). Moreover, higher differences in the RAVLT immediate recall during the first learning trial were trend-to-significant related to higher interim ATS use (*n* = 17, *r* = −0.44, *P* = 0.075).

## Discussion

Due to its prospective longitudinal design and the recruitment of occasional ATS users with very limited ATS exposure at study inclusion, the present study enabled a specific assessment of ATS use associated brain morphological changes. Importantly, users in the LOW and HIGH groups did not differ regarding previous or interim use of frequently co-used drugs, including cannabis and alcohol, which often present severe confounders in the field and may affect striatal functional and structural integrity (Gouzoulis-Mayfrank and Daumann, 2009; Zhou *et al*., 2019; Zimmermann *et al*., 2019; Grodin and Momenan, 2017). Together with the findings on a dose-response relationship in the correlational analyses, that took advantage of the entire sample, this suggests a direct association between the use of ATS and decreased dorsal striatal GM volumes and cognitive performance. Memory deficits, particularly immediate and delayed verbal memory, have been among the most consistently reported neurocognitive changes in ATS users, including chronic MA users (Scott *et al*., 2007; Roberts *et al*., 2018) as well as non-dependent populations such as recreational MDMA (Wagner *et al*., 2013; Schilt *et al*., 2007) and prescription AMPH users (Reske *et al*., 2010). However, despite the consistently observed functional impairments in the memory domain, and associated hippocampal memory functioning (Becker *et al*., 2013) evidence for altered structural hippocampal volume as a consequence of occasional or chronic ATS use is rather equivocal (Ersche *et al*., 2013; Daumann *et al*., 2011; Mackey *et al*., 2014; Berman *et al*., 2008). This may be may be related to the methodological properties of the VBM approach. Alterations in memory-related hippocampal functioning of ATS users are thought to due to changes in serotonergic (5HT) functioning (Wagner *et al*., 2013; Schilt *et al*., 2007). However, whereas previous studies that combined in vivo receptor PET and VBM indicate a strong positive association between regional GM volume and dopaminergic D2/D3 receptor binding (Woodward *et al*., 2009), associations between 5HT receptor distribution and regional GM volume have not been reported (e.g. Jedema *et al*., 2010), suggesting that longitudinal VBM might have a higher sensitivity to detect alterations in DA pathways.

In line with the longitudinally observed associations between occasional ATS use and GM changes, previous cross-sectional studies revealed some evidence for brain-structural effects of occasional ATS use. A large study in occasional ATS and cocaine users revealed increased putamen and decreased inferior parietal GM volumes in occasional users as compared to non-using controls (Mackey and Paulus, 2013). In contrast, a previous cross-sectional comparison made by our group between baseline data from the occasional ATS users in the present study and drug-naïve controls did not reveal alterations in GM volume, probably due to the low-dose ATS exposure at study inclusion (Daumann *et al*., 2011). In addition, several cross-sectional studies examined brain morphological markers of chronic ATS use in MA-dependent individuals. Comprehensive reviews and meta-analytic evaluations of these cross-sectional comparisons revealed accumulating evidence for a consistent pattern of decreased prefrontal GM volumes accompanied by increased dorsal striatal, particularly putamen volumes in chronic MA users relative to controls (Ersche *et al*., 2013; Mackey and Paulus, 2013; Mackey *et al*., 2014). Several studies reported that within the group of MA users, increased putamen volume was inversely associated with cognitive dysfunction (Chang *et al*., 2005; Jernigan *et al*., 2005; Jan *et al*., 2012), suggesting that increasing the GM volume of the striatum may be a compensatory response to initial neurotoxic effects.

In contrast to the consistently observed increases in putamen GM volume in chronic ATS users, we found a decreased volume in continuing low-dose ones. Taken together with the association between GM and verbal memory decline, this might suggest that compensatory responses have not yet occurred in the present sample. One study examined effects of short-term ATS exposure on cognitive functioning and brain-structural markers in children who were exposed to MA prenatally (Chang *et al*., 2004) . In line with the present findings, children with MA exposure demonstrated relative reductions in striatal, including putamen, volume, and cognitive deficits in the domains of attention and memory. Notably, verbal memory deficits were specifically associated with the volume of the globus pallidus and the putamen (Chang *et al*., 2004).

Studies examining the effects of ATS use at the molecular level have repeatedly observed deficient dorsal striatal DA neurotransmission in chronic MA users associated with functional deficits in motor and memory functioning (Volkow *et al*., 2001; Taylor *et al*., 2013). Likewise, controlled studies in nonhuman primates observed decreased markers of dopaminergic functioning in the putamen following escalating MA regimes (Groman *et al*., 2013) as well as dopaminergic deficits in the dorsal striatum after low-dose AMPH exposure (Ricaurte *et al*., 2005). Notably, an escalating MA regimen caused regionally specific increased GM volumes in the putamen (Groman *et al*., 2013). Together with the previously reported correlation between regional GM volume and dopaminergic functioning (Woodward *et al*., 2009) this might suggest that the present findings parallel altered DA functioning in the dorsal striatum as a consequence of ATS use.

Although the dopaminergic basal ganglia (BG) system has been traditionally implicated in motor functioning and procedural learning (Bonelli and Cummings, 2007; Robbins *et al*., 2008) more recent evidence from BG disorders, particularly from Parkinson disease (PD), lesion studies and pharmacological neuroimaging studies, has revealed that the dorsal striatum contributes to learning and memory (Ward *et al*., 2013; Grahn *et al*., 2009). Cognitive impairments, most consistently in the domains of learning and memory, are a well-recognized feature in the early stages of PD (Grahn *et al*., 2009). Dopaminergic deficits in the putamen present the primary pathology during these initial stages of the disorder (Rodriguez-Oroz *et al*., 2009; Owen *et al*., 1998) and functional impairments show an extreme sensitivity to DA modulation (Lange *et al*., 1992), suggesting that they have a primary DA substrate. In addition, loss of putamen volumes has specifically been associated with cognitive deterioration in other neurodegenerative disorders characterized by marked memory impairments, including Alzheimer's disease (de Jong *et al*., 2008). Moreover, evidence from patients with focal lesions to the BG, including the putamen, revealed impairments in the cognitive domains of working and verbal memory (Ward *et al*., 2013). One prospective study examining brain structure and cognitive functioning in 73 patients after carbon monoxide poisoning reported that stronger verbal memory impairments were associated with smaller putamen volumes 6 months following poisoning (Pulsipher *et al*., 2006). Recently, putamen volume has been genetically associated with schizophrenia, which is a psychiatric disorder characterized by its cognitive deficits (Luo *et al*., 2019). Although our knowledge of the role of the putamen in cognitive functioning is still incomplete these findings, together with the present results, indicate that alterations to its structure and function may result in more substantial cognitive impairment than previously assumed.

Although the present prospective longitudinal design allowed to control for several important confounders inherent to retrospective design, the findings have to be interpreted in the context of several limitations. First, several of the participants did not participate in the follow-up assessment and thus the sample size is comparably low and the findings need to be replicated in larger populations. Second, the study protocol and the target regions were not pre-registered and thus the findings should be considered as exploratory. Third, longer follow-up periods are necessary to determine the maintenance or recovery of the cognitive and brain functional changes over longer abstinence periods.

In summary, the present study has provided the first longitudinal evidence that prolonged use of low-dose ATS is associated with decreased dorsal striatal GM volume and verbal memory deficits, possible reflecting alterations in DA functioning.
